# Research, Education, Workforce, and Regulation to Transforming Nursing Practice and Leadership: The Conundrum of “Where to Start”

**DOI:** 10.3390/healthcare11030378

**Published:** 2023-01-29

**Authors:** Arianna Magon, Alessandro Stievano, Irene Baroni, Rosario Caruso

**Affiliations:** 1 Health Professions Research and Development Unit, IRCCS Policlinico San Donato, San Donato Milanese, 20097 Milano, Italy; 2 Centre of Excellence for Nursing Scholarship, OPI, 00173 Rome, Italy; 3 Department of Clinical and Experimental Medicine, University of Messina, 98100 Messina, Italy; 4 Department of Biomedical Sciences for Health, University of Milan, 20133 Milano, Italy

Transforming nursing practice and leadership is an ongoing effort in the nursing profession, and it involves several key components, such as research, education, workforce, and regulation [1,2,3,4]. The starting point is, “why do we have to transform nursing practice and leadership?”. Even if the question might appear self-evident, the reasons supporting a bold change of direction in the way nurses are able to express their full potential in healthcare, socio-economic processes, sciences, and, broadly, culture, are powerful, rational, and well-rooted in the literature, but these reasons seem unheard or misjudged in many ways [5].

Triggering virtuous cycles in nursing research, education, workforce, and regulations is key to improving patient outcomes and nurses’ well-being, soundly supporting the changing healthcare landscape, addressing healthcare demands and unmet needs, supporting the integration of technology and innovation in patient care, realizing patient-centered care beyond slogans, and disseminating a culture of health in addressing ongoing global challenges [6]. Triggering virtuous cycles means that improving one aspect of the nursing profession, such as regulations, research, or education, will lead to improvements in other areas, such as clinical practice, workforce capacity, and conditions. However, starting a virtuous cycle is a complex matter. In this editorial, we would like to add our thoughts about the need to trigger virtuous cycles to support nursing in expressing its full potential in order to create a roadmap for the near future, which has been, and is, extensively requested in several forms of scientific, professional, and social organizations [7]. However, the prospects must be more extensively discussed and understood in several levels of decision-making and policies globally.

Overall, triggering the above-mentioned virtuous cycles requires a comprehensive approach to improving patient outcomes, supporting nurses’ well-being, and addressing the ongoing global challenges in healthcare. Assuming that addressing one aspect of the nursing profession can lead to improvements in other areas is valid for creating a positive feedback loop. In this case, the challenge is “where to start,” and this challenge is also antecedent to the most common question of “how to start”. In general, there are several possible starting points, depending on the contextual scenario shaped by healthcare characteristics, epidemiological distribution of healthcare needs, labor arena, regulatory and socio-economic factors, and cultural aspects.

Starting from research could be strategic, because investing in nursing research can lead to discovering and/or implementing new and effective nursing care, best practices, and academic engagement that can improve patient- and system-level healthcare outcomes. Starting from research could develop new educational programs and improve existing ones, which can lead to better-educated and more skilled nurses, and a positive feedback loop has a chance to start. The contexts that may benefit from beginning from research are likely those with an established university education for nurses and embracing self-regulation to fulfill the need for public protection.

Investing in nursing education can also lead to developing new and improved educational programs to better prepare nurses to meet the changing healthcare landscape. This approach could be suited for contexts moving toward strengthening university-based education, postgraduate specializations, and advanced practice. This possible starting point can lead to an improved nursing workforce, associated with better patient outcomes, and, again, it seems possible to start a virtuous cycle here.

It is likely that investing in the nursing workforce could lead to the recruitment and retention of highly qualified nurses, leading to improved patient- and system-related outcomes. This approach could lead to the development of new and improved educational programs, better salaries, and a more attractive nursing profession, which can lead to better-educated and more skilled nurses competing to contribute to high-quality healthcare delivery. This scenario fits the contexts with issues in attractiveness for nursing careers that wish to start a positive feedback loop in the healthcare ecosystem.

Investing in regulation can lead to the development of new policies, standards, and guidelines for broadening the scope of nursing practice that can improve patient outcomes by fulfilling the need for public protection and meeting unmet healthcare demands. Nursing regulations and public protection are closely linked, as regulation plays an important role in ensuring that nurses meet the standards of practice and provide safe, high-quality care to the public [8]. More precisely, nursing regulations cover aspects related to the standards of practice, licensure (i.e., the process of granting a license to practice nursing), continuing education, professional conduct, ethics, and accountability. Investing in regulations, in turn, could lead to increased public trust and confidence in the nursing profession. This can advance social recognition of the role of nurses in healthcare. This starting point seems suited for contexts where it is required to increase social recognition and broaden the scope of nursing practice toward advanced roles, consequently triggering structured postgraduate education and new positions for advanced roles in the labor market.

Triggering virtuous cycles in nursing research, education, workforce, and regulation can be started in many ways, and this approach should be tailored to the healthcare system’s and community’s specific needs and challenges. For example, if the healthcare system faces a shortage of nurses (as this is a current global issue), one starting point could be investing in the nursing workforce. This approach could involve recruiting and retaining highly qualified nurses and improving working conditions, which could lead to improved patient outcomes. This loop can advance the development of new and enhanced educational programs and the improvement of existing ones, leading to better-educated and more skilled nurses.

Another example can be related to primary care challenges [9]. If the healthcare system is facing weaknesses in primary care services, one starting point could be investing in nursing research focused on primary care. This approach could bring the definition of new and effective primary care interventions and models that can improve patient outcomes and the way the system meets healthcare needs, which in turn can lead to the development of new educational programs and the improvement of existing ones. This loop can be conducive to better-educated and more skilled nurses in the primary care field. Changing the challenge of the example: if the healthcare system faces integrating technology and innovation in patient care, one starting point could be investing in education programs to train nurses in the use of technology, advancing their digital literacy, and innovating patient care [10,11]. This can lead to better patient outcomes, the development of new and improved educational programs, and so on, with a positive feedback loop.

Triggering virtuous cycles in nursing research, education, workforce, and regulation can lead to a better healthcare system that is more sustainable, patient-centered, and innovative. It seems that the conundrum of “where to start,” which we have discussed in this editorial, leads us to conclude that it is possible to start from every angle and with multiple strategies. The important message we wish to share is that there is an urgent need to shift from rhetoric to factual starting of sustainable initiatives to trigger the virtuous cycles that every healthcare system worldwide needs.

## References

[1] Osingada C.P., Porta C.M. (2020). Nursing and Sustainable Development Goals (SDGs) in a COVID-19 World: The State of the Science and a Call for Nursing to Lead. Public Health Nurs..

[2] Kunaviktikul W., Turale S. (2020). Internationalizing Nursing Curricula in a Rapidly Globalizing World. Nurse Educ. Pract..

[3] Rosser E.A. (2022). Investing in Nursing and Its Leadership to Secure Global Health: Are We Making Progress?. Int. Nurs. Rev..

[4] Catton H. (2023). Nursing Our Planet. Int. Nurs. Rev..

[5] Rasheed S.P., Younas A., Mehdi F. (2020). Challenges, Extent of Involvement, and the Impact of Nurses’ Involvement in Politics and Policy Making in in Last Two Decades: An Integrative Review. J. Nurs. Sch..

[6] (2019). The Lancet 2020: Unleashing the Full Potential of Nursing. Lancet.

[7] World Health Organization (2020). State of the World’s Nursing 2020: Investing in Education, Jobs and Leadership.

[8] Stievano A., Caruso R., Pittella F., Shaffer F.A., Rocco G., Fairman J. (2019). Shaping Nursing Profession Regulation through History—A Systematic Review. Int. Nurs. Rev..

[9] Dellafiore F., Caruso R., Cossu M., Russo S., Baroni I., Barello S., Vangone I., Acampora M., Conte G., Magon A. (2022). The State of the Evidence about the Family and Community Nurse: A Systematic Review. Int. J. Environ. Res. Public Health.

[10] Conte G., Caruso R., Della Fiore F., Magon A., Ghizzardi G., Arrigoni C. (2022). Mappatura Della Letteratura Sulle Soluzioni Digitali e Tecnologiche Nell’assistenza Infermieristica: Un Protocollo Di Scoping Review. Prof. Inferm..

[11] Caruso R., Arrigoni C., Conte G., Rocco G., Dellafiore F., Ambrogi F., Stievano A. (2021). The Byzantine Role of Big Data Application in Nursing Science: A Systematic Review. CIN Comput. Inform. Nurs..

